# Language Choices at Home and Their Relationship With Educational Outcomes, With a Special Focus on Children With Origins in Former Yugoslavia and Turkey in Six European Countries

**DOI:** 10.3389/fsoc.2022.841847

**Published:** 2022-06-15

**Authors:** Elina Kilpi-Jakonen, Jenni Alisaari

**Affiliations:** ^1^INVEST Research Flagship Centre, University of Turku, Turku, Finland; ^2^Faculty of Education and Welfare Studies, Applied Pedagogy, Åbo Akademi University, Turku, Finland

**Keywords:** children of immigrants, language maintenance and language shift, reading scores, educational expectations, school climate, school composition

## Abstract

Language has been conceptualized as both a measure as well as a predictor of integration among immigrants and their children. However, the relationship between language spoken at home and different educational outcomes remains poorly understood. Many studies indicate that nurturing students' first languages is positively associated with their learning at school. Other research suggests that one of the reasons why children of immigrants tend to perform worse at school is due to speaking a language other than that of instruction at home. In order to shed further light on the role of language choices at home for education, we examine both the correlates of language use at home as well as the relationship between this and reading scores and educational expectations. We differentiate between three language use groups: those who mainly use the language of origin at home, those who only use the language of instruction at home, and those who use both of these. We analyze these relationships using data from the Programme for International Student Assessment (PISA). In order to examine country differences, we place a special focus on two immigrant-origin groups that are present in significant numbers in a number of European countries: children with origins in Turkey and former Yugoslavia. These two groups have also been identified as being at major educational disadvantage across Europe. Our results suggest that continuing to (mainly) use the language of origin at home is more prevalent among children from socioeconomically more disadvantaged families, but is supported by more socioeconomically advantaged and more diverse school environments. In the majority of countries studied, switching to the language of instruction is associated with higher reading scores but not with higher educational expectations than continuing to speak mainly the language of origin at home. These relationships are to a large extent confounded (or in some cases potentially mediated) by family factors such as socioeconomic status and school-related factors such as school's socioeconomic composition. We conclude by highlighting the role that linguistically responsive pedagogies and a positive school climate can play for the education of all young people but in particular newly-arrived immigrants.

## Introduction

Many studies indicate that nurturing students' first languages is positively associated with their learning of other languages and subjects at school as well as their educational expectations (see e.g. Cummins, 2001; Ovando and Combs, 2011; Feliciano and Lanuza, 2016; Agirdag and Vanlaar, 2018; Ganuza and Hedman, 2018). Speaking a language other than the language of instruction at home is, nevertheless, not without challenges. For example, some studies show that language spoken at home is a significant predictor of an achievement gap between immigrant background students and their native peers (Dustmann et al., 2012). The challenges students face at school are greater when the distance between the first language and the language of instruction is wider (Floccia et al., 2018; Borgonovi and Ferrara, 2020). However, using a language other than the language of instruction *per se* does not prevent students from learning the language of instruction or result in worse learning outcomes (Strobel, 2016). On the contrary, strong first language skills benefit the learning of the language of instruction as well as other subjects (Cummins, 2001; Ganuza and Hedman, 2018).

The language spoken at home by immigrants and their children has been used as both a measure of integration as well as a predictor of integration. Nevertheless, the relationship between language spoken at home and other dimensions of integration remains poorly understood. Students with migrant origins are at a higher risk of early school leaving and lower academic outcomes compared to their native peers (OECD, 2015), and a lack of host-country relevant linguistic, cultural, relational, and informational resources has been found to be associated with lower educational opportunities for children of immigrants (Borgna, 2017). Yet disadvantages tend to remain even when controlling for socioeconomic factors and parents' cultural capital, though these vary depending on both the country of destination and origin (Heath et al., 2008). In this article we aim to explore in more detail the role that language spoken at home plays for the educational outcomes of children of immigrants, in terms of both learning outcomes (in particular reading scores) and expectations, and how this may depend on the country of destination. We examine the relationship of family- and school-related factors with language use patterns at home, and whether these factors confound (or potentially mediate) the relationship between language use and educational outcomes, in other words whether there is a direct relationship.

For immigrant youth, schools play an important role in the integration process. According to previous research, school climate has a strong impact on how immigrant background students succeed at school and how they experience a sense of belonging in their school (e.g., Schachner et al., 2019). Therefore, in addition to school composition, we also examine whether differences in school climate play a role for explaining differences in educational outcomes between language use groups.

Our main research questions are thus:

1. How are family and school-related characteristics associated with continuing to speak mainly the language of parental origin (L1) at home, on the one hand, and with switching to the language of the country of residence, in particular that of the school (second language/L2), on the other?
2. How are patterns of language use associated with reading test scores and educational expectations? Do parental resources and school characteristics confound these associations?

In order to study these question cross-nationally, we place a special focus on two immigrant-origin groups that are present in significant numbers in a number of European countries: children of immigrants with origins in Turkey and former Yugoslavian countries (see e.g., Veermlan and Dronkers, 2016). These two groups have also been identified as being at a major educational disadvantage across Europe (e.g., Crul, 2013; Schnell and Fibbi, 2016). Thus, our third research question is:

3. Are there country differences in these associations? Can any of these differences be understood through country differences in institutions or policies?

## Previous Research and Context

### Languages in Immigrant Families

The role of language for immigrants' wellbeing and integration is far from simple. Language proficiency is essential for becoming socialized into the linguistic and cultural behaviors of different communities (Phinney and Ong, 2007), and this applies to both co-ethnic (and transnational) communities (Hulsen et al., 2002; Parameshwaran, 2014; Soehl, 2016) as well as the majority community within the country of destination, enabling structural integration (Chiswick and Miller, 2002; Bleakley and Chin, 2004; Glick et al., 2013). Language is also an important part of one's identity; a positive orientation to both one's own culture (of origin) as well as toward the culture of the settlement country are important for psychological wellbeing (Berry et al., 2006).

Theories of segmented assimilation have identified the maintenance of the culture and language of the country of origin as an alternative pathway to integration, also providing children of immigrants with a route to achieving high educational attainment (Portes and Rumbaut, 2006). In contrast, theories of straight-line assimilation predict that over time and across generations, immigrants cease to use their language of origin and switch to using the language of the country of destination (Alba and Nee, 2003). Research examining language maintenance and shift tends to find relatively rapid shifts across generations in L2 use and proficiency and corresponding L1 attrition (e.g., Alba et al., 2002; Parameshwaran, 2014; Soehl, 2016). Nevertheless, contextual and group differences as well as life-cycle variation exists (e.g., Tran, 2010; Pauwels, 2015).

Learning a new language also takes time. Academic language that is required for studying different subjects at school differs notably from everyday language (Cummins, 2000). Everyday language develops generally within 2 years (Cummins, 1979), but at least 4–8 years is required for students to reach the level of their native peers in academic language proficiency (Collier, 1987). The age of arrival to the new country is a significant predictor of learning the language of instruction: students who arrive at ages 8–11 have been found to have an advantage in terms of the time it takes to develop academic language compared to their peers that arrive earlier (5–7 years old) or especially to ones who arrive later (12–15 years old) (Collier, 1987).

From an intergenerational perspective, language use can be an intentional choice made by parents and which relates to both their own and their children's integration. There are parents who see L2 acquisition as so important that they decide to switch family language (Chatzidaki and Maligkoudi, 2013). However, not all families are in a position to make an active choice related to family language policy, particularly when parents themselves do not speak the L2. Better educational resources and labor market incorporation of parents are likely to increase their possibilities of supporting their children's L2 acquisition, but also of enhancing their children's L1 skills. Previous research has found that children of more highly educated parents tend to be less likely to speak the L1 at home (e.g., Alba et al., 2002; Chiswick and Gindelsky, 2016). At the same time, highly educated parents are also more likely to raise children who are fluent bilinguals (Portes and Hao, 2002; Portes and Rumbaut, 2006; Soehl, 2016). Indeed, high levels of proficiency (native-likeness) in L1 and L2 can go hand-in-hand (Bylund et al., 2012).

Languages are learnt in social interaction, mediated by other language users within the surrounding environment (Vygotsky, 1978; Van Lier, 2000; Lantolf, 2007), and schools form an essential platform for learning languages. At the level of the community (including neighborhood and schools), the ethnic/linguistic composition has been found to influence children of immigrants' use of and proficiency in L1, whereby a larger proportion of co-ethnics is associated with a higher likelihood to continue speaking the L1 and a higher proficiency in it (Alba et al., 2002; Portes and Rumbaut, 2006; Rydland et al., 2013; van Tubergen and Mentjox, 2014; Chiswick and Gindelsky, 2016). This may come through an influence on both the formal language learning environments (provision of formal teaching of the L1 in schools, availability of weekend schools) as well as the environment for informal language learning and use (see also Rydland et al., 2013).

### Languages and Educational Outcomes

A number of studies have shown that bilingualism (or L1 use) is beneficial for children of immigrants in the US for various educational outcomes (grades, high school completion, and educational expectations) when compared to being proficient in (or using) L2 only (Feliciano, 2001; Glick and White, 2003; Lutz and Crist, 2009; Agirdag, 2014; Feliciano and Lanuza, 2016). There is also cross-national evidence that language minority students who speak their L1 more often with their parents have at least equal educational performance with those who speak the L2 more often at home, with those speaking the L1 at home outperforming their L2-speaking peers in some countries (Agirdag and Vanlaar, 2018).

Much of the theoretical reasoning behind these beneficial effects has dealt with social capital formation either within the family (Lutz and Crist, 2009; Oh and Fuligni, 2010) or linking to the co-ethnic group (Feliciano, 2001; Portes and Hao, 2002). In particular, the segmented assimilation approach argues that selective acculturation, where children and parents continue holding on to elements of their country of origin's culture, including its language, and are supported in this by a strong co-ethnic community, can provide children with an alternative route to high educational and labor market attainment, despite their parents' low human capital (e.g., Portes and Rumbaut, 2006). However, recent research from Germany testing two mechanisms based on hypotheses around L1 use at home leading to more social capital and more effective use of social capital and thus mediating the relationship between L1 use at home and better educational outcomes, did not find support for either of these two hypotheses (Strobel, 2016).

Children with a migration background have been found to have higher educational aspirations and expectations in a wide variety of country contexts (e.g., Chykina, 2019; Rudolphi and Salikutluk, 2021). This has often been found to result from so-called immigrant optimism and in particular from high parental aspirations (Teney et al., 2013; Feliciano and Lanuza, 2016; Salikutluk, 2016; Tjaden and Hunkler, 2017). The ability of parents to transmit these aspirations to their children can in turn be dependent on family cohesion as well as continued use of the L1 at home. Feliciano and Lanuza (2016) found that L1 use between parents and children (measured during kindergarten) explained the higher educational expectations of children of immigrants in the US that were still evident after controlling for parental expectations and the children's own interest in school work. In the context of Norway, Friberg (2019) found language use to explain some of the immigrant advantage in “idealistic” educational aspirations though not for “realistic” educational expectations.

Another explanation that has been put forward for these higher educational aspirations and expectations particularly in the European context has been that children of immigrants (and their parents) lack proper information about how the education system works (e.g., Salikutluk, 2016; Tjaden and Hunkler, 2017). If this is the case then this may also translate as higher expectations for children who use the L1 at home if we assume that one reason for doing so is that their parents (and potentially the children themselves) are less fluent in L2 and thus are likely to have lower levels of information (cf. Mouw and Xie, 1999). This may also be more influential in countries with more stratified educational systems.

Research evidence from cognitive science also supports the view that maintenance of the language of origin (in the form of bilingualism) provides benefits in terms of cognitive functioning (Kroll et al., 2015; Bialystok and Grundy, 2018). In a meta-analysis of the literature, Adesope et al. (2010) found that bilinguals outperformed monolinguals on measures of metalinguistic and metacognitive awareness as well as measures of abstract and symbolic representation, attentional control, and problem solving. However, a more recent meta-analysis suggests that at least some of these results may be due to publication bias and that bilingualism is not associated with superior executive functioning (Lehtonen et al., 2018).

At the same time, it should be acknowledged that different L1s are not equally valued across countries of settlement (see also Pendakur and Pendakur, 2002). In other words, whereas some immigrants may have an L1 that is also valued among the majority population, others' L1 may be seen by the majority population as being of value only within the co-ethnic community (or country of origin). This may influence how L1 maintenance links to other outcomes. For example, in Sweden a strong attachment to L1 and ethnic identity has been found to be associated with the most positive incorporation outcomes in terms of psychological and socio-cultural adaptation for Turkish-origin youth (Vedder and Virta, 2005). This is in contrast to the Netherlands, where the policy focus has shifted strongly to an emphasis on L2 acquisition, and where the best incorporation outcomes for Turkish-origin youth also come from strong attachment to L2 and a non-ethnic identity (Vedder and Virta, 2005). In contrast, for young people of Surinamese origin in the Netherlands, better L1 proficiency has even been found to be associated with lower psychological and sociocultural adaptation (Vedder, 2005). Indeed, there is also research suggesting that speaking a language other than that of instruction is associated with poorer school performance (Schnepf, 2007; Dustmann et al., 2012). For example, it has been found that family background and language spoken at home explain the gap in learning outcomes between immigrant background students and their native peers entirely in Germany, and over 60% of the gap in Austria, Belgium and Switzerland (Dustmann et al., 2012). However, the duration of the stay in the host country is also found to be a significant factor affecting the learning outcomes (Böhlmark, 2008; Ohinata and Van Ours, 2012), which is also linked to language acquisition.

Nevertheless, there is a broader argument about the need for L2 learners to continue developing their L1s and for students' whole linguistic repertoire to be used as a resource since languages are interdependent (Cummins, 2001, 2021). Research has documented how monolingual (L2) practices in schools are detrimental to the educational achievement of language-minority students (e.g., Thomas and Collier, 1997; García and Hesson, 2015). Furthermore, students' L1 skills are connected with their L2 skills (Edele and Stanat, 2016), and thus, promoting and developing L1 skills might positively influence also L2 skills and the learning of other school subjects (Cummins, 2021). Taking immigrant-origin students' linguistic backgrounds into consideration in educational settings can take various forms: teaching may take place in the L1 (or bilingually), L1 may be taught as a separate subject, and pedagogical practices may be linguistically responsive. Research suggests that bilingual educational programs are more beneficial for students than instruction only in the majority language (Ramirez, 1991; Thomas and Collier, 1997), and that students benefit from attending L1 lessons (Ganuza and Hedman, 2018). However, in terms of resources required, the first of these requires the most and it is also the most restricted in the number of students it is practical to reach since bilingual educational programs can only ever be suitable when there is a very large concentration of students from the same linguistic backgrounds. The second of these, teaching of immigrant-origin students' L1 as a separate subject, is regulated or recommended in 13 European education systems, particularly in many Northern European countries (Eurydice, 2019). Nevertheless, even in these countries not all students receive L1 teaching, for example due to regulations on the minimum number of interested students. The last of these (i.e., linguistically responsive pedagogies) has the potential to reach all students from multilingual backgrounds since it is not tied to any specific language. Language awareness has been included as a transversal competence in the education systems of Brandenburg (Germany), Austria, and Finland (Eurydice, 2019). In the first two systems, this relates in particular to the language of instruction, whereas in Finland the core curriculum stresses plurilingualism for all learners. However, even in Finland teachers do not necessarily have sufficient knowledge and skills on how to implement pedagogies that would support students' use of their L1s (Alisaari et al., 2019).

### School Climate

School climate can be defined as the environment that a school provides, and it includes factors such as safety, relationships and its mission (Cohen et al., 2009). According to a review by Cohen et al. (2009), a positive school climate is safe, caring, participatory, and encouraging, and it seems to be associated with a range of positive outcomes including academic achievement, students' healthy development, and teacher retention. The participatory nature of the school culture is highly valuable: The more co-operative the school culture is, the better learning outcomes the students tend to have (OECD, 2019b). According to Govorova et al. (2020), the way students perceive the school climate explains both their social engagement and their anxieties. When students perceive that the school climate values diversity and intercultural communication, they seem to have a higher sense of school belonging which in turn seems to be associated with better outcomes (Schachner et al., 2019). Notably, a positive school and intergroup climate has been found to be related to better school outcomes for immigrant minority youth (Celeste et al., 2019; Schachner et al., 2019; Berkowitz, 2022).

School safety has been found to be associated with higher levels of students' belief in self, consisting of self-efficacy, persistence, and self-awareness, which in turn seem to be associated with higher levels of school engagement (Storlie and Toomey, 2020). On the contrary, declines in perceived school climate have been shown to be unidirectionally associated with declines in psychological and behavioral adjustment of students (Way et al., 2007). According to previous studies conducted in US, it seems that there are significant differences in experiences of school climate depending on both individual-level factors such as gender, ethnicity and parent's education (Fan et al., 2011), as well as school-level measures such as ethnic composition or students' SES and academic performance (Jain et al., 2015).

Appropriate teaching strategies can contribute to the positive development of school climate (Govorova et al., 2020). Moreover, positive interactions between teachers and students promote an inclusive climate at school (Mælan et al., 2020) and students' wellbeing (Eccles and Roeser, 2011; Suldo et al., 2012; Mannion et al., 2015; Anderson and Graham, 2016). Especially teachers' support and the way they maintain classroom disciplinary climate seem to have a central role in students' attitudes toward school and their sense of belonging (Chiu et al., 2012). Since being part of a group is one of people's basic needs (Baumeister and Leary, 1995), the feeling of belonging is related to the perception that a person is accepted as a member of a group (Lambert et al., 2013). A school climate that creates a feeling of belonging as well as a sense of being respected and listened to, supports individual students' wellbeing (Anderson and Graham, 2016). More broadly, students' perceptions of school (Allodi, 2010; Aldridge et al., 2018) and classroom climate (Eccles and Roeser, 2011) are associated with their sense of wellbeing and life-satisfaction.

A school atmosphere that is positive, participatory and supporting co-operation, and where students have a strong sense of belonging, may be assumed to increase the acceptance and inclusion of students' diverse linguistic and cultural backgrounds. Supporting positive diversity climate is of utmost importance in diverse schools: According to a study by Rjosk et al. (2017), a high proportion of ethnic minority students or ethnically diverse classrooms are connected with students' lower sense of belonging as well as lower educational outcomes of individual students. Other studies have shown no effect of the proportion of natives or co-ethnics at the school on school performances in secondary education for Turkish origin students, and a negative association between ethnic diversity and learning outcomes in math, possibly because of lesser opportunities to gain access to social networks of native peers (Veermlan and Dronkers, 2016). However, also contradictory results exist: In the Netherlands, Peetsma et al. (2006) found that a greater amount of migrants in a class was positively associated with math scores for students from Turkish and Moroccan origin, and they explain this result with the expertise that teachers have gained in adapting their teaching according to students' needs (see also Veermlan and Dronkers, 2016).

It should also be noted that ethnic diversity is often closely correlated at the school level with socioeconomic disadvantage, which may be the stronger compositional feature influencing school performance (Fekjær and Birkelund, 2007; Cebolla-Boado and Garrido Medina, 2011). However, instead of the ethnic minority composition it might be more relevant to look at the proportion of minority language students at school, namely school's ethno-lingual composition, as a factor influencing students' academic achievement (Seuring et al., 2020). The role of language in school achievements can be explained by aspects related to quality of instruction, school resources, or organizational issues and interaction with peers (Thrupp et al., 2002; see also Seuring et al., 2020). Quality of instruction and support is essential in order to reach good learning results, especially for vulnerable learners and language learners (Tharp et al., 2000; Carrasco, 2014; Harju-Autti et al., 2021). In addition, if the school has a number of students who share the same minority language, they may support each other's learning through their first languages (Seuring et al., 2020).

Based on these theoretical premises, we examine how school composition is associated with language use with parents, with the assumption that a more diverse school in terms of linguistic minorities is also associated with greater acceptance of minority language use and thus also increased use of L1 at home with parents. In addition, we examine how a broader set of school climate measures, which include students' perceptions of co-operation within schools and their sense of belonging to school—measured at both the individual and the school level—are associated with educational outcomes and whether they can explain differences between the language use groups. This latter may come about if the different language groups tend to attend different types of schools or if they perceive the school climate differently even when attending the same schools.

### Country Contexts

In this article, we focus on Austria, Belgium, Denmark, Germany, Luxembourg, and Switzerland. These countries have sufficiently detailed information about the students' and their parents' countries of birth as well as the language spoken at home in the data used (PISA-2018, OECD, 2019a) for us to analyze language use patterns. In addition, they have large enough numbers of students with origins in former Yugoslavia and Turkey to enable meaningful analyses—though students with origins in former Yugoslavia cannot be distinguished as a separate group in Belgium and students with origins in Turkey cannot be distinguished as a separate group in Luxembourg.

Education systems vary in many ways. One way to define differences is to look at the levels of tracking in different parts of the education paths. If the level of tracking is high, the education system has multiple education programs for children of the same age and the programs are hierarchically ranked (Bol and van de Werfhorst, 2013). Often the academic track prepares students into tertiary education, while vocational education prepares them earlier into the labor market (Lessard-Phillips et al., 2014). In Austria, Belgium, Germany, Luxembourg and Switzerland, there is an early selective system at the lower secondary stage into academic and vocational schooling, whereas in Denmark, the selection happens much later (Heath et al., 2008). Highly tracked education systems seem to increase inequality and reduce equal opportunities, which means that social origins are more strongly associated with educational performance and attainment (Bol and van de Werfhorst, 2013). For example, early stratification has been found to lead to greater disadvantages for the second generation (Schnell, 2014).

Austria, Belgium, Germany, Luxembourg, and Switzerland have highly stratified education systems. Based on the school success, teachers' recommendations, and (in some countries) parents' will, students are streamed into separate types of school already at the early age: in lower-secondary education at the age of ten in Austria (Schnell, 2014), at the age of 12 in Belgium (Baysu and de Valk, 2012) and in Luxembourg (Hoffmann, 1998), at the age between ten and twelve in Switzerland (Fibbi et al., 2015), and, even though the different streams of education start at the secondary level, the decision concerning the educational path is mainly done at the age of nine or ten in Germany (Worbs, 2003). Although there are possibilities to change tracks later during the educational path in Austria and Germany, the first selection might hold through the whole education pathway (Worbs, 2003; Lessard-Phillips et al., 2014; Schnell, 2014). In Belgium and Switzerland, there is not much movement between different tracks (Lessard-Phillips et al., 2014). In Switzerland, after compulsory education, students may either continue to higher secondary education (consisting of different options) leading to tertiary education, or vocational training, and also tertiary education has different forms that have varied requirements concerning previous studies (Fibbi et al., 2015). In Belgium, all educational programs allow attending university (Baysu and de Valk, 2012). In Denmark, 9 years of compulsory education is followed by upper secondary education, which is divided into academic and vocational programmes, stratification to tertiary education or to the labor market happens after upper secondary education (Egelund, 2005).

In the studied countries, students with an L1 other than the language of instruction, are offered instruction in their L1 in various ways: According to Eurydice (2009), in French speaking Belgium, L1 instruction has been available at ISCED levels 1 and 2, in other words in primary and lower secondary education, whereas in Dutch speaking Belgium it is restricted to ISCED level 1 (primary education). In Denmark, L1 instruction covers ISCED levels 1 and 2, but is offered only in EU languages, as well as Faroese and Greenlandic (Timm and Kristjánsdóttir, 2011). In the German school system, there is also a possibility to attend mother tongue instruction after regular classes (c.f. Worbs, 2003). L1 lessons are often held outside normal school hours, either in afternoons or even on Saturdays like in some cantons in Switzerland (Kanton Zürich, 2021). In Luxembourg and Austria, they are often included in the mainstream school curriculum (Eurydice, 2009).

### Children of Immigrants From Turkey and Former Yugoslavia in Western Europe

In post-World War II Western Europe, economic growth rapidly created a call for migrant workers, for example from Turkey, which experienced population growth and unemployment at the same time, leading to a large Turkish-origin population in Germany, Austria, the Netherlands, France, Belgium, and Sweden (Schnell, 2014; Zuccotti et al., 2017). In Belgium, France, Germany, the Netherlands, and Switzerland this migration was supported through formal intergovernmental guest-worker programs, and it was assumed to be temporary (Heath et al., 2008). Later this migration continued as family reunions, especially in Germany, Austria, and France (Schnell, 2014; Zuccotti et al., 2017). In the 1970s, refugees (mainly Kurdish, Assyrian, or Syriani) started to flee from Turkey to Western Europe (Schnell, 2014). At the moment, people with Turkish origins are the largest immigration group in Europe (Veermlan and Dronkers, 2016).

In the 1960s and 1970s, after Yugoslavia changed its emigration policies (Fibbi et al., 2015), there started to be an important labor emigration toward Germany, northern Europe and Switzerland, partly through similar guest-worker programs (Heath et al., 2008). In the 1990s emigration from former Yugoslavia continued as family reunifications and through asylum seeking (Heath et al., 2008; Fibbi et al., 2015). These migrations have led to notable second generation groups with Turkish or former Yugoslavian backgrounds in the aforementioned countries (Heath et al., 2008).

Children of immigrants with origins in Turkey and former Yugoslavian countries have been identified as being at a major educational disadvantage in a number of European countries (e.g., Crul, 2013; Schnell and Fibbi, 2016), as well as having “stigmatized cultural backgrounds” (Heikamp et al., 2020, p. 781 for Turkish origin students, Fibbi et al., 2015 for people with former Yugoslavian backgrounds). Further, these groups are among the largest immigrant groups in many countries (Phalet et al., 2007; Veermlan and Dronkers, 2016; Heikamp et al., 2020), and share other characteristics, as well: both groups consist mainly of migrant workers and their families, but also refugees (Fibbi et al., 2015).

Among the second generation, children with Turkish origins seem to be the most disadvantaged group, at least in Austria, Belgium, Denmark, Germany, the Netherlands, Norway, Sweden, and Switzerland (Worbs, 2003; Heath et al., 2008; Dustmann et al., 2012; Fibbi et al., 2015). Additionally, even though adolescents from the former Yugoslavia tend to do slightly better compared to their peers with Turkish origins, their school situation is concerning as well (Heath et al., 2008; Dustmann et al., 2012; Fibbi et al., 2015). There are ethnic-specific penalties for students of Turkish origins, especially for the first generation (Kalter and Granato, 2007), but the disadvantage is often transmitted and cumulated to the second generation through perpetual ethnic educational inequalities in schools (Phalet et al., 2007). The educational gap between children from Turkish origins and their majority peers is wide and it is related to their socioeconomic backgrounds, but also to the concentration of immigrant pupils in certain schools, which has been found to affect the school climate, the motivation of teachers, and language acquisition (Timmerman et al., 2003). Children with Turkish origins are more likely than majority children to attend a school with a lower proportion of majority children, which may be due to parents' lack of knowledge of school systems and possible alternatives (Kristen, 2008). Spending time with native peers would be, however, beneficial for the children of Turkish immigrants, whereas segregated schools can limit the probability of continuing to secondary education (Crul and Schneider, 2009). In fact, a smaller network of majority peers, along with developing language skills, may explain migrant penalties for children with Turkish origins (Kalter, 2006). Migrants with Turkish origins don't have as developed majority language skills compared to other migrant groups, e.g., those from the former Yugoslavia, and this continues in the second generation (Diehl and Schnell, 2006). This may have an influence on their education, too, as reflected in PISA results (Heath et al., 2008).

Nevertheless, educational expectations in these groups, particularly among young people with a Turkish origin, tend to be high, at least when taking into account their social origin and school performance. Turkish origin students at around age 14 have been found to be more likely to hold university aspirations than their majority peers in Sweden, Germany, and the Netherlands and the same holds for the former Yugoslav origin group in Sweden but not in Germany (Rudolphi and Salikutluk, 2021). The same holds for Turkish origin youth in Brussels (Teney et al., 2013). Examined from the perspective of vocational educational training, Turkish origin youngsters in Germany have been found to be less inclined to intend to apply for dual training (apprenticeship) than their majority peers (Tjaden and Hunkler, 2017).

The educational attainment of the second generation with Turkish origin benefits from starting school at an early age and not having an early stratification to a special track, and especially entering kindergarten at the age of two or three is effective (Crul and Schneider, 2009). Many Turkish-origin children repeat one or more years at primary school, and even in the second generation, many students spend some time at school in Turkey which may have a negative influence on their education (Timmerman et al., 2003). Even though the second generation moves up the educational hierarchy compared to the people living in Turkey and to their own parents, they still do not reach the level of Western European majority peers in education or in the labor market (Dustmann et al., 2012; Zuccotti et al., 2017), and given their educational level, they have lower educational returns in Western Europe than they would have in Turkey (Zuccotti et al., 2017).

In many of the countries that are the focus of this study, students with immigrant backgrounds are overrepresented in vocational tracks. In Austria, along with parents' education level, the early selection seems to be a crucial explanation for the education orientation of the Turkish second generation (Schnell, 2014). In Germany, adolescents with Turkish or Yugoslavian origins attend more frequently vocational training compared to their majority peers, and they also enter the labor market earlier (Worbs, 2003). Similarly in Switzerland, Turkish origin adolescents are over-represented in elementary requirement tracks compared to those from Serbia-Montenegro (Fibbi et al., 2015). Overall, in Germany, when the second generation's educational level is compared to their parents' education, the Turkish-origin children often remain at the similar (low) educational level with their parents, although the proportion of upward mobility is higher than of their majority peers (Worbs, 2003). The Former-Yugoslavian origin children also are mainly stable with their educational level compared to their parents, but a remarkably high proportion are downwardly mobile (Worbs, 2003).

Students' educational paths can be negatively affected by discrimination as well. A Turkish background is related to a high stigmatization for example in Belgium (Heikamp et al., 2020), and Turkish-origin youth who had experienced discrimination or stereotype threat, often disengage from academic tasks (Baysu et al., 2016). In Switzerland, the second generation of Turkish origin report significantly higher levels of hostility toward them than those with former Yugoslavian backgrounds, and the context of this hostility is often the school, with teachers and other staff members reported as being hostile (Fibbi et al., 2015). When individuals feel belonging to a certain group that experiences discrimination, they might perceive social identity threat leading to focusing on failing rather than possibilities to succeed since they base part of their self-concept on the group (Derks et al., 2007). Nevertheless, according to recent studies Turkish-origin adolescents tend to have high levels of integration whereby they have a positive attitude toward both the majority society as well as their own ethnic group (Diehl and Schnell, 2006, see also Berry, 1997). Discrimination does not seem to decrease the level of integration (Jugert et al., 2020). In Switzerland, despite the educational challenges, second-generation students with Turkish or Former Yugoslavian origins perceive the school system as giving everybody equal opportunities (Fibbi et al., 2015).

In Belgium, Austria and Germany, majority group friends increase the probability of children of Turkish (and Moroccan) immigrants to attend educational tracks that lead to university (Baysu and de Valk, 2012). In Austria, second-generation Turks evaluate their peers' effect on their educational outcomes to be even more important than that of their parents (Schnell, 2014). The ethnic composition of the peers matter significantly: having plenty of native friends as close friends positively influences educational attainment, and the effect of native peers is higher than parents' education levels or parental support (Schnell, 2014).

## Materials and Methods

The research is carried out using the most recent wave of the Programme for International Student Assessment (PISA- 2018; OECD, 2019a). As mentioned above, we focus on the following OECD countries: Austria, Belgium, Denmark, Germany, Luxembourg, and Switzerland. We divide children of immigrants (both parents born abroad) into three groups based on whether they report speaking a language other than that of instruction at home and the extent to which they speak that language with their parents (mothers and fathers asked separately). This results in three groups: (1) those who mainly speak the L1 at home with both parents, (2) those who speak a mixture of L1 and L2 with their parents, and (3) those who speak only the L2 at home with their parents. We also separate the so-called 2.5 generation, i.e., those with one foreign-born and one native-born parent, and the fifth category is composed of majority students (two native-born parents). These two latter categories of students are only included in the analyses of educational outcomes.

Only the 2012 and 2018 PISA assessments include the detailed questions about language use at home, whereas in other years only the question of whether another language (than that of the test) is used at home is included. However, PISA-2012 did not include other measures of key interest here and thus we use only the 2018 data.

A special focus is placed on students whose both parents (or one for the second set of analyses) were born in the former Yugoslavia or in Turkey. Former Yugoslavia includes slightly different parts of the region in the different destination countries but we aim to include all students born in the Balkans. Supplementary Tables include results where these two groups are separated.

Our dependent variables for the first research question are mainly speaking the L1 at home in contrast with the two other language groups, and switching completely to the L2 at home in contrast with continuing to speak the L1 at least to some extent. Supplementary Tables include results where the dependent variable is speaking both the L1 and L2 with parents. For this research question we only include children of immigrants (no native born parents) in our models.

Our dependent variables for the second research question are reading scores and educational expectations. PISA-2018 focuses on literacy, and thus the literacy measurements can be considered to be more accurately measured in 2018 data set than the other two competencies (math and science). Language use at home is also likely to influence literacy results to a greater extent than math results, and it thus requires critical attention. Supplementary Tables also include results for math scores. Educational expectations are measured with a question of whether the student expects to complete university-level education (ISCED 5A or 6).

For the second research question we also include the 2.5 generation and majority students in our sample. We have also replicated all the analyses excluding the majority students. These results can be found in the Supplementary Tables.

Our independent variables of interest for the first research question are generation (1st generation, i.e., born abroad, vs. the rest), age at arrival for the first generation (defined as 0 for all others), and parental SES (the OECD's index of economic, social and cultural status) at the individual level. Given that all students in PISA are 15 years old, the influence of age at arrival cannot be separated from length of residence, which should be kept in mind when interpreting the results. At the school level we focus on school mean SES and proportion of foreign-language students with migrant origins (1st and 2nd generation). We also tested the proportion of students from one's own national origin, but the results seem to be more closely related to the overall proportion of foreign language migrant origin students. This may, however, be due to greater measurement error in the proportion from one's own national origin than in the proportion of foreign language students with migrant origins.

For the second research question we also add two measures related to school climate. These are the perception of co-operation at school and sense of belonging to school. These are measured by three questions for perception of co-operation (Students seem to value cooperation; It seems that students are cooperating with each other; Students seem to share the feeling that cooperating with each other is important) and six questions for sense of belonging to school (I feel like an outsider (or left out of things) at school; I make friends easily at school; I feel like I belong at school; I feel awkward and out of place in my school; Other students seem to like me; I feel lonely at school). There are four answer categories for all of these. We use the OECD-constructed standardized scales. We include both of these at both the individual level and aggregated at the school level. We also tested scales measuring perception of competition and being bullied, but the results were stronger with the two scales that were chosen.

Our control variables in all models are gender, student's grade in comparison with the modal grade for 15 year-olds in the respective countries, and language of instruction at school, which is relevant for Belgium, Luxembourg, and Switzerland. For the first research question we also control for country of (parental) origin in all models, and for the second research question we add this in the second model. We use as detailed countries of origin as possible within all the countries analyzed. If parents were born in two different foreign countries we use that of the mother, except when we focus on children with origins in Turkey and former Yugoslavia, when we include all children with at least one parent born in these countries, regardless of whether this is the mother or the father.

We use listwise deletion for missing values. There was a great deal of missingness for the scales related to school climate in particular. Therefore, the samples are different for the first research question, where these scales are not used, and the second one. Nevertheless, there are relatively small differences in the proportion of the three language groups among children of immigrants between these two samples. However, it should be noted that the samples for the second research question are slightly more advantaged than those for the first, for example in terms of parental SES and competence scores, in particular in Germany, which displays the most missingness for the measures related to school climate. Table 1 displays the original number of students in the data and the sample sizes for the two research questions for each country. The lower panel also gives descriptive statistics for all the variables used, except for language groups, which are in Table 2 and described more fully below.

**Table 1:** Descriptive information of samples and variables.

	**AUT**	**BEL**	**DNK**	**GER**	**LUX**	**SWI**	**Total**
Original *N* (all)	6,802	8,475	7,657	5,451	5,230	5,822	39,437
*N* for RQ1 (all)	1,345	1,361	1,406	987	2,643	1,895	9,637
*N* for RQ2 (all)	5,027	6,789	5,745	2,076	4,199	3,290	27,126
**Individual level variables (based on sample for RQ2)**							**Range**
Parental SES	0.064	0.155	0.432	0.032	0.089	0.044	−6.727, 3.890
	(0.853)	(0.898)	(0.820)	(1.007)	(1.117)	(0.912)	
First generation	0.057	0.067	0.026	0.038	0.228	0.108	0/1
Age at migration (1st gen)	6.889	7.441	6.303	7.551	7.109	7.186	0, 16
Gender (female)	0.513	0.513	0.508	0.473	0.500	0.472	0/1
Relative grade	−0.518	−0.310	−0.139	0.538	0.370	0.315	−3, 3
	(0.579)	(0.572)	(0.378)	(0.648)	(0.664)	(0.613)	
Perception of co-operation at school	0.374	−0.006	0.301	0.091	−0.032	0.139	−2.143, 1.676
	(1.006)	(0.973)	(0.873)	(1.022)	(1.022)	(1.021)	
Sense of belonging to school	0.446	0.082	0.219	0.338	0.111	0.285	−3.258, 3.234
	(1.242)	(0.892)	(1.038)	(1.038)	(1.015)	(1.045)	
Reading	497.3	508.4	500.4	524.0	483.4	489.2	159.9, 777.6
	(92.2)	(92.5)	(86.8)	(98.6)	(102.0)	(98.3)	
Math	509.2	521.7	505.6	521.9	494.4	518.2	202.3, 767.0
	(83.0)	(84.3)	(75.2)	(86.9)	(88.8)	(85.7)	
Expectation to complete HE	0.364	0.480	0.582	0.358	0.516	0.388	0/1
**School level variables (based on sample for RQ2)**
School SES	0.058	0.145	0.413	0.009	0.055	0.025	−1.891, 1.330
	(0.434)	(0.457)	(0.392)	(0.547)	(0.622)	(0.450)	
School proportion FL immig. (centered at 0.1)	0.047	−0.004	−0.014	0.027	0.293	0.097	−0.100, 0.900
	(0.159)	(0.124)	(0.115)	(0.140)	(0.178)	(0.155)	
School perception of co-op.	0.365	−0.007	0.297	0.085	−0.044	0.132	−2.143, 1.676
	(0.342)	(0.412)	(0.285)	(0.389)	(0.208)	(0.437)	
School sense of belonging	0.422	0.072	0.205	0.315	0.094	0.271	−3.237, 2.725
	(0.307)	(0.249)	(0.256)	(0.284)	(0.214)	(0.311)	

*Standard deviations in parentheses*.

**Table 2:** Distribution of language use groups (%) among all children of immigrants (upper panel) and youth with origins in Turkey and former Yugoslavia (lower panel).

**All children of immigrants**	**Austria**	**Belgium**	**Denmark**	**Germany**	**Luxembourg**	**Switzerland**
Mainly L1	47.0	35.3	26.2	34.6	64.7	41.4
Both L1 and L2	25.4	18.1	17.6	26.6	6.6	19.6
Only L2	27.6	46.6	56.2	38.8	28.8	39.0
*N*	1,368	1,310	1,424	1,005	2,393	1,870
**Selected**	**Austria**	**Belgium**	**Denmark**	**Germany**	**Luxembourg**	**Switzerland**
Mainly L1	48.2	44.5	22.9	30.4	85.7	47.3
Both L1 and L2	30.3	30.3	19.4	35.9	7.4	30.4
Only L2	21.5	25.2	57.7	33.7	6.9	22.3
*N*	799	112	273	273	245	676

We use multilevel linear regression models that are run separately by country, meaning that for the binary dependent variables these are linear probability models. The multilevel models take into account the clustering of students in schools (random intercepts), which is particularly important when estimating the school-level effects. We estimate the models using Stata 16's mixed command. The OECD's weighting procedure in PISA combines weights at the level of the school and the individual student, which produces biased estimates in the multilevel framework that we use. Thus, the results that we present are without weights. However, the models have also been run taking into account the stratification of the sample by using the recommended weighting procedure but with clustered standard errors at the level of the school rather than with a multilevel model. The results of these models were largely consistent with the results presented here. For the analyses of reading (and math) scores, we run the models with the ten plausible values using Stata 16's multiple imputation tools.

## Results

### Language Maintenance and Loss

We begin by examining the predictors of language maintenance, in particular mainly speaking the L1 with parents, and language switching. Table 2 shows the proportion of students in each of our three language groups in the analyzed countries, in the upper panel for all children of immigrants and in the lower panel for our selected subgroup. It should be noted that in the case of all children of immigrants, not all of those who report speaking only the L2 at home are what we would consider language switchers: they may come from countries where the language of destination is the majority language and thus their parents' language of origin. This is likely to be the case particularly in Belgium, Luxembourg, and Switzerland.

The results in this table suggest that switching to L2 is most common in Denmark, where 56–58% of both samples report speaking only Danish with their parents and only 23–26% report speaking mainly the L1. At the other extreme is Luxembourg, where 65% of all children of immigrants and 86% of our subgroup report speaking mainly the L1. The other countries are in-between these two extremes, with Germany somewhat closer to Denmark, and Austria, Belgium and Switzerland remarkably similar when we examine children with origins in former Yugoslavia and Turkey. In these countries, 44–48% speak mainly the L1, 21–25% switch to L2, and 30% mix both languages.

Table 3 displays the results for the models predicting speaking mainly the L1 with parents. At the family level, both parental SES and generation as well as age at migration are significant predictors of continuing to speak mainly the L1 in most countries. Both of the examined school level factors also seem to be associated with strong L1 maintenance, though not necessarily in the expected directions.

**Table 3:** Predictors of speaking mainly the L1 with parents among all (upper panel) and selected group (lower panel).

**All**	**Austria**	**Austria**	**Belgium**	**Belgium**	**Denmark**	**Denmark**	**Germany**	**Germany**	**Lux**.	**Lux**.	**Swi**.	**Swi**.
	**M1a**	**M2a**	**M1a**	**M2a**	**M1a**	**M2a**	**M1a**	**M2a**	**M1a**	**M2a**	**M1a**	**M2a**
SES	**−0.09**	**−0.10**	**−0.05**	**−0.05**	*−0.03*	**−0.05**	**−0.06**	**−0.06**	**−0.04**	**−0.04**	**−0.04**	**−0.04**
	(0.02)	(0.02)	(0.01)	(0.01)	(0.01)	(0.01)	(0.01)	(0.02)	(0.01)	(0.01)	(0.01)	(0.01)
1st gen.	0.09	0.08	0.06	0.06	*0.11*	*0.12*	**0.17**	**0.17**	*0.04*	*0.04*	**0.16**	**0.16**
	(0.05)	(0.05)	(0.04)	(0.04)	(0.05)	(0.05)	(0.06)	(0.06)	(0.02)	(0.02)	(0.04)	(0.04)
Age at migr.	*0.01*	*0.01*	**0.02**	**0.01**	**0.03**	**0.03**	**0.03**	**0.03**	0.00	0.00	**0.02**	**0.02**
	(0.00)	(0.00)	(0.00)	(0.00)	(0.01)	(0.01)	(0.01)	(0.01)	(0.00)	(0.00)	(0.00)	(0.00)
% FL migr.		**0.24**		**0.50**		**0.68**		**0.44**		*0.16*		**0.41**
		(0.09)		(0.10)		(0.09)		(0.11)		(0.10)		(0.08)
School SES		*0.08*		*0.08*		**0.15**		**0.09**		−0.02		**0.13**
		(0.04)		(0.04)		(0.04)		(0.03)		(0.03)		(0.03)
*N*	1,345	1,345	1,361	1,361	1,406	1,406	987	987	2,643	2,643	1,895	1,895
**Selected**	**Austria**	**Austria**	**Belgium**	**Belgium**	**Denmark**	**Denmark**	**Germany**	**Germany**	**Lux**.	**Lux**.	**Swi**.	**Swi**.
	**M1b**	**M2b**	**M1b**	**M2b**	**M1b**	**M2b**	**M1b**	**M2b**	**M1b**	**M2b**	**M1b**	**M2b**
SES	**−0.11**	**−0.11**	**−0.20**	**−0.19**	*−0.05*	*−0.06*	**−0.11**	**−0.10**	*−0.05*	*−0.05*	**−0.06**	**−0.07**
	(0.02)	(0.02)	(0.05)	(0.04)	(0.02)	(0.02)	(0.02)	(0.03)	(0.02)	(0.02)	(0.02)	(0.02)
1st gen.	0.13	0.13	−0.02	−0.02	*0.26*	*0.24*	**0.43**	**0.42**	0.05	0.05	0.11	0.11
	(0.07)	(0.07)	(0.29)	(0.27)	(0.12)	(0.12)	(0.15)	(0.15)	(0.08)	(0.08)	(0.08)	(0.08)
Age at migr.	0.01	0.01	0.03	0.03	*−0.06*	*−0.06*	0.02	0.02	−0.01	−0.01	0.01	0.01
	(0.01)	(0.01)	(0.03)	(0.03)	(0.02)	(0.02)	(0.01)	(0.01)	(0.01)	(0.01)	(0.01)	(0.01)
% FL migr.		0.23		**1.16**		**0.67**		0.12		0.33		0.38*
		(0.12)		(0.26)		(0.18)		(0.19)		(0.37)		(0.15)
School SES		0.04		0.11		*0.19*		0.00		0.08		0.08
		(0.06)		(0.11)		(0.07)		(0.06)		(0.12)		(0.06)
*N*	800	800	120	120	276	276	284	284	296	296	683	683

*Multilevel linear probability models; reference category for first generation is second generation; all models control for gender, grade, language of the school, and country of origin; bold p < 0.01, italics p < 0.05*.

Going into more detail, the higher parental SES is, the lower the probability of continuing to speak mainly the L1. The relationship is similar for both all children of immigrants as well as Turkish and ex-Yugoslav subgroup. Nevertheless, there are indications that the association is stronger among this subgroup than among other children of immigrants, particularly in Belgium and Germany. The first generation tends to be more likely to continue speaking mainly the L1 even when comparing those who arrived at a very young age to those who were born in the destination country. This is particularly the case in Denmark, Germany and Switzerland, whereas the difference is substantially smaller and not significant in Austria and Belgium, in Luxembourg the difference is the smallest but nevertheless statistically significant. More or less the same is the case among the Turkish and ex-Yugoslav subgroup although in Switzerland the difference is smaller and not statistically significant. As age at migration increases (and length of residence decreases), the probability to continue speaking mainly the L1 also increases among all children of immigrants except in Luxembourg. Among our subgroup, this relationship also tends to be slightly weaker although the main difference that stands out is in Denmark, where a later age at migration is actually associated with a lower probability to continue speaking mainly the L1.

At the school level the proportion of foreign-language immigrant-origin speakers is positively associated with the probability to mainly speak the L1 in all countries, although in Luxembourg the estimate is not quite as large as in most of the countries and not statistically significant. There is more variation when it comes to the association among the Turkish and ex-Yugoslav subgroup: in Austria, Belgium, Denmark and Switzerland the estimate is at least as large as for all children of immigrants (though not statistically significant in Austria) whereas it is smaller and not significant in Germany (and slightly larger but still not significant in Luxembourg). Somewhat surprisingly, being in a school with higher SES peers is associated with a greater likelihood of speaking mainly L1 with parents in Austria, Belgium, Denmark, Germany, and Switzerland—though it should be remembered that this is after controlling for all the other covariates in the model. Among our subgroup of interest, this relationship only holds in Denmark, whereas in all other countries the estimates are small and not significant.

In our supplementary analyses we replicate these analyses with children with origins in the Former Yugoslavia and in Turkey separately. On the whole, the results are relatively similar and the samples are in any case slightly too small to draw any firm conclusions about differences between the groups. The one difference that seems to be relatively consistent, however, is that family SES tends to be slightly more strongly associated with speaking mainly the L1 with parents among those with Turkish origins.

Table 4 then displays the results for having switched to using the L2 with parents. As can be expected, the results largely mirror those presented previously, though with some minor variations. For example, in Austria, Belgium, and Germany the association with parental SES is not quite as strong. This concerns both all children of immigrants and our selected subgroup, for whom this is also the case in Luxembourg. The difference between early childhood migrants and the second generation, with the first generation being less likely to switch to L2, is also mainly present in Germany and Switzerland for all children of immigrants but not statistically significant for our subgroup. In fact, the relationship is in the opposite direction among the Turkish and ex-Yugoslav subgroup in Belgium and Luxembourg, though only the latter is statistically significant. The results for age at migration tend to be similar as earlier, with later arrival (shorter length of residence) being associated with a lower likelihood to switch. Among the selected subgroup, the earlier exception of Denmark continues (later arrival associated with a greater likelihood to switch) but the estimate is not statistically significant.

**Table 4:** Predictors of speaking only the L2 with parents among all (upper panel) and selected group (lower panel).

**All**	**Austria**	**Austria**	**Belgium**	**Belgium**	**Denmark**	**Denmark**	**Germany**	**Germany**	**Lux**.	**Lux**.	**Swi**.	**Swi**.
	**M1c**	**M2c**	**M1c**	**M2c**	**M1c**	**M2c**	**M1c**	**M2c**	**M1c**	**M2c**	**M1c**	**M2c**
SES	**0.05**	**0.06**	**0.03**	**0.03**	*0.03*	**0.04**	*0.03*	0.03	**0.03**	**0.02**	*0.03*	**0.03**
	(0.01)	(0.01)	(0.01)	(0.01)	(0.01)	(0.01)	(0.01)	(0.01)	(0.01)	(0.01)	(0.01)	(0.01)
1st gen.	−0.05	−0.05	0.02	0.00	−0.11	−0.11	**−0.16**	**−0.16**	−0.03	−0.03	**−0.16**	**−0.16**
	(0.04)	(0.04)	(0.04)	(0.04)	(0.06)	(0.06)	(0.06)	(0.06)	(0.02)	(0.02)	(0.03)	(0.03)
Age at migr.	*−0.01*	**−0.01**	**−0.02**	**−0.01**	**−0.02**	**−0.02**	**−0.02**	**−0.02**	*−0.00*	−0.00	*−0.01*	*−0.01*
	(0.00)	(0.00)	(0.00)	(0.00)	(0.01)	(0.01)	(0.01)	(0.01)	(0.00)	(0.00)	(0.00)	(0.00)
% FL migr.		**−0.38**		**−0.58**		**−1.16**		**−0.73**		*−0.21*		**−0.71**
		(0.07)		(0.09)		(0.10)		(0.11)		(0.09)		(0.08)
School SES		**−0.11**		**−0.10**		**−0.27**		−0.09*		0.04		**−0.13**
		(0.03)		(0.03)		(0.04)		(0.03)		(0.03)		(0.03)
*N*	1,345	1,345	1,361	1,361	1,406	1,406	987	987	2,643	2,643	1,895	1,895
**Selected**	**Austria**	**Austria**	**Belgium**	**Belgium**	**Denmark**	**Denmark**	**Germany**	**Germany**	**Lux**.	**Lux**.	**Swi**.	**Swi**.
	**M1d**	**M2d**	**M1d**	**M2d**	**M1d**	**M2d**	**M1d**	**M2d**	**M1d**	**M2d**	**M1d**	**M2d**
SES	**0.05**	**0.06**	*0.10*	*0.10*	0.05	*0.06*	**0.08**	0.05	0.03	0.02	**0.08**	**0.08**
	(0.02)	(0.02)	(0.04)	(0.04)	(0.03)	(0.03)	(0.03)	(0.03)	(0.01)	(0.02)	(0.02)	(0.02)
1st gen.	−0.06	−0.05	0.41	0.40	−0.17	−0.11	−0.27	−0.25	*0.12*	*0.11*	−0.09	−0.10
	(0.06)	(0.06)	(0.26)	(0.25)	(0.15)	(0.14)	(0.17)	(0.17)	(0.05)	(0.05)	(0.07)	(0.07)
Age at migr.	−0.01	−0.01	*−0.06*	*−0.06*	0.02	0.01	−0.01	−0.01	*−0.02*	*−0.02*	0.00	−0.00
	(0.01)	(0.01)	(0.03)	(0.03)	(0.03)	(0.03)	(0.02)	(0.02)	(0.01)	(0.01)	(0.01)	(0.01)
% FL migr.		**−0.41**		*−0.62*		**−1.53**		−0.19		−0.14		**−0.70**
		(0.10)		(0.30)		(0.21)		(0.22)		(0.18)		(0.13)
School SES		*−0.10*		−0.12		**−0.32**		0.08		0.01		−0.10
		(0.05)		(0.13)		(0.09)		(0.07)		(0.06)		(0.06)
N	800	800	120	120	276	276	284	284	296	296	683	683

*Multilevel linear probability models; reference category for first generation is second generation; all models control for gender, grade, language of the school, and country of origin; bold p < 0.01, italics p < 0.05*.

The school-level associations also mirror the previous ones and are in some cases even stronger: the proportion of foreign-language immigrant-origin students at the school is inversely related to the probability of switching to L2 in all countries, with the relationship being particularly strong in Denmark. Among the selected subgroup, the relationship is somewhat weaker and not statistically significant in Germany and Luxembourg. School peers' SES is also inversely related to the probability of switching in all countries except Luxembourg, and the Turkish and ex-Yugoslav subgroup tends to be quite similar (though not always statistically significant) except in Germany, where the relationship is the opposite, though again not statistically significant.

Analyzing the two groups separately does not indicate that any of the results reported would be solely due to patterns in one of the groups rather than the other.

Results for speaking both L1 and L2 with parents (in the Supplementary Materials) show, as expected largely a mix of results. In Austria, Germany and Luxembourg, a more advantaged parental SES is associated with a greater likelihood to speak both languages, approximately comparable to the association with speaking the L2 only. In Belgium this is also the case for the selected subgroup. Interestingly, the school composition results, especially the proportion of foreign-language immigrant-origin students, tend to follow the ones related to speaking mainly the L1, though less strongly.

### Home Language, Schools, and Educational Outcomes

We then move to the analysis of how patterns of language use at home are related to educational outcomes. Table 5 displays the language group estimates for reading scores from three models: M1 with just the basic controls, M2 adding parental SES, migration generation and age at arrival, as well as the country of origin, and M3 adding the school-related measures. Table 6 does the same for educational expectations. Since the results for the school-related measures are of interest in their own right, the results for these variables (M3) are displayed for both outcomes in Table 7 but only for the full sample.

**Table 5:** Language group differences in reading scores among all (upper panel) and selected group (lower panel).

**All**	**AUT**	**AUT**	**AUT**	**BEL**	**BEL**	**BEL**	**DNK**	**DNK**	**DNK**	**GER**	**GER**	**GER**	**Lux**	**Lux**	**Lux**	**Swi**	**Swi**	**Swi**
	**M1a**	**M2a**	**M3a**	**M1a**	**M2a**	**M3a**	**M1a**	**M2a**	**M3a**	**M1a**	**M2a**	**M3a**	**M1a**	**M2a**	**M3a**	**M1a**	**M2a**	**M3a**
Both L1 and L2	−0.47	−3.10	−2.64	12.63	10.82	10.93	**−21.46**	*−21.11*	*−20.93*	2.12	−5.21	−3.63	5.61	−2.75	−1.45	1.13	−4.51	−2.79
	(5.82)	(5.85)	(5.81)	(6.62)	(6.73)	(6.72)	(8.08)	(8.31)	(8.28)	(10.49)	(10.90)	(10.75)	(7.17)	(7.26)	(7.22)	(6.91)	(7.01)	(6.96)
Only L2	**22.37**	**15.37**	**16.79**	**26.45**	**21.56**	**22.46**	0.59	−0.59	−0.58	**34.33**	20.60	19.27	**36.72**	**24.12**	**23.67**	**24.18**	11.77	*13.07*
	(5.52)	(5.64)	(5.61)	(5.42)	(5.90)	(5.87)	(6.36)	(6.80)	(6.80)	(10.16)	(10.91)	(10.80)	(4.84)	(5.24)	(5.21)	(5.74)	(6.07)	(6.03)
2.5 gen	**39.07**	**23.95**	**24.99**	**30.97**	**21.65**	**22.11**	**37.60**	13.17	12.62	*21.06*	9.32	10.55	**22.37**	**12.15**	**12.43**	**37.97**	*15.68*	**17.69**
	(4.92)	(5.60)	(5.58)	(4.82)	(5.68)	(5.68)	(6.19)	(7.52)	(7.56)	(9.14)	(10.19)	(10.13)	(3.91)	(4.51)	(4.50)	(5.17)	(6.61)	(6.59)
Majority	**50.51**	**53.44**	**52.81**	**39.14**	**23.89**	**25.38**	**52.46**	13.13	12.00	**37.38**	*25.82*	*24.40*	**34.77**	**24.41**	**23.89**	**48.71**	**28.52**	**29.88**
	(4.05)	(5.99)	(5.98)	(4.38)	(6.29)	(6.29)	(5.36)	(10.04)	(10.10)	(8.13)	(10.08)	(10.09)	(3.69)	(5.62)	(5.61)	(4.82)	(6.67)	(6.68)
Family controls		x	x		x	x		x	x		x	x		x	x		x	x
School controls			x			x			x			x			x			x
*N*	5,027	5,027	5,027	6,789	6,789	6,789	5,745	5,745	5745	2,076	2,076	2,076	4,199	4,199	4,199	3,290	3,290	3,290
**Selected**	**AUT**	**AUT**	**AUT**	**BEL**	**BEL**	**BEL**	**DNK**	**DNK**	**DNK**	**GER**	**GER**	**GER**	**Lux**	**Lux**	**Lux**	**Swi**	**Swi**	**Swi**
	**M1b**	**M2b**	**M3b**	**M1b**	**M2b**	**M3b**	**M1b**	**M2b**	**M3b**	**M1b**	**M2b**	**M3b**	**M1b**	**M2b**	**M3b**	**M1b**	**M2b**	**M3b**
Both L1 and L2	1.38	−0.25	0.71	20.79	4.07	7.69	−15.17	−16.99	−14.91	10.40	7.02	5.22	−12.12	−16.58	−15.61	6.55	1.09	1.83
	(6.85)	(6.87)	(6.82)	(18.23)	(18.61)	(18.44)	(18.42)	(18.26)	(18.22)	(17.49)	(19.19)	(18.85)	(17.29)	(17.30)	(17.15)	(9.71)	(9.70)	(9.57)
Only L2	13.75	12.11	*15.00*	20.97	6.77	11.39	−7.70	−4.00	−2.15	**68.25**	**63.07**	**52.78**	37.85	33.68	31.02	*21.00*	13.50	13.21
	(7.44)	(7.41)	(7.38)	(19.13)	(19.43)	(19.20)	(15.34)	(15.48)	(15.54)	(18.05)	(19.89)	(19.50)	(24.56)	(24.40)	(24.17)	(10.14)	(10.11)	(9.93)
2.5 gen	**24.59**	**21.64**	**22.80**	26.30	11.49	15.13	−18.71	−20.41	−17.37	**42.30**	33.96	27.77	36.68	34.23	33.22	23.01	5.96	6.39
	(7.88)	(7.83)	(7.78)	(14.24)	(14.74)	(14.57)	(17.22)	(17.57)	(17.59)	(15.92)	(17.80)	(17.55)	(20.37)	(20.38)	(20.16)	(12.97)	(13.26)	(13.17)
Majority	**53.74**	**45.52**	**46.66**	**64.15**	**39.90**	**38.25**	**51.34**	5.85	6.75	**75.46**	**61.88**	*47.48*	**37.12**	**27.51**	**27.08**	**60.10**	**37.55**	**35.91**
	(4.82)	(5.40)	(5.44)	(11.47)	(12.55)	(12.44)	(13.25)	(13.90)	(13.96)	(14.09)	(19.56)	(19.20)	(6.20)	(7.00)	(7.00)	(7.27)	(7.85)	(7.92)
Family controls		x	x		x	x		x	x		x	x		x	x		x	x
School controls			x			x			x			x			x			x
*N*	4,254	4,254	4,254	4,947	4,947	4,947	4,259	4,259	4,259	1,582	1,582	1,582	1,530	1,530	1,530	1,999	1,999	1,999

*Multilevel linear models with ten plausible values; reference category: speaking mainly L1; all models control for gender, grade, and language of the school; M2 additionally controls for generation, age at migration, country of origin and parental SES; M3 additionally controls for proportion foreign language migrant origin in school, mean SES in school, perception of co-operation and sense of belonging at both individual and school levels (see Table 7); bold p < 0.01, italics p < 0.05*.

**Table 6:** Language groups differences in educational expectations among all (upper panel) and selected group (lower panel).

**All**	**AUT**	**AUT**	**AUT**	**BEL**	**BEL**	**BEL**	**DNK**	**DNK**	**DNK**	**GER**	**GER**	**GER**	**Lux**	**Lux**	**Lux**	**Swi**	**Swi**	**Swi**
	**M1c**	**M2c**	**M3c**	**M1c**	**M2c**	**M3c**	**M1c**	**M2c**	**M3c**	**M1c**	**M2c**	**M3c**	**M1c**	**M2c**	**M3c**	**M1c**	**M2c**	**M3c**
Mix	−0.01	−0.04	−0.04	−0.02	−0.05	−0.04	−0.07	*−0.10*	*−0.10*	*−0.14*	*−0.14*	*−0.12*	0.03	−0.02	−0.01	**−0.10**	**−0.12**	**−0.12**
	(0.03)	(0.03)	(0.03)	(0.04)	(0.04)	(0.04)	(0.04)	(0.04)	(0.04)	(0.06)	(0.06)	(0.06)	(0.04)	(0.04)	(0.04)	(0.04)	(0.04)	(0.04)
Switch	0.03	0.00	0.02	−0.00	−0.03	−0.02	−0.05	*−0.08*	*−0.08*	−0.05	−0.08	−0.07	0.04	−0.03	−0.03	*−0.06*	**−0.12**	**−0.11**
	(0.03)	(0.03)	(0.03)	(0.03)	(0.03)	(0.03)	(0.03)	(0.04)	(0.04)	(0.05)	(0.06)	(0.06)	(0.03)	(0.03)	(0.03)	(0.03)	(0.03)	(0.03)
2.5	0.02	−0.05	−0.02	−0.05	**−0.08**	*−0.07*	−0.03	**−0.13**	**−0.12**	−0.06	**−0.15**	*−0.13*	0.04	−0.03	−0.02	−0.03	**−0.15**	**−0.14**
	(0.03)	(0.03)	(0.03)	(0.03)	(0.03)	(0.03)	(0.03)	(0.04)	(0.04)	(0.05)	(0.05)	(0.05)	(0.02)	(0.03)	(0.03)	(0.03)	(0.03)	(0.03)
Maj.	0.02	**−0.13**	**−0.12**	**−0.10**	**−0.19**	**−0.16**	−0.04	**−0.24**	**−0.23**	−0.07	**−0.21**	**−0.19**	0.03	−0.03	−0.04	*−0.06*	**−0.23**	**−0.21**
	(0.02)	(0.03)	(0.03)	(0.02)	(0.04)	(0.04)	(0.03)	(0.06)	(0.06)	(0.04)	(0.05)	(0.06)	(0.02)	(0.03)	(0.03)	(0.02)	(0.03)	(0.03)
Family controls		x	x		x	x		x	x		x	x		x	x		x	x
School controls			x			x			x			x			x			x
*N*	5,027	5,027	5,027	6,789	6,789	6,789	5,745	5,745	5,745	2,076	2,076	2,076	4,199	4,199	4,199	3,290	3,290	3,290
**Selected**	**AUT**	**AUT**	**AUT**	**BEL**	**BEL**	**BEL**	**DNK**	**DNK**	**DNK**	**GER**	**GER**	**GER**	**Lux**	**Lux**	**Lux**	**Swi**	**Swi**	**Swi**
	**M1d**	**M2d**	**M3d**	**M1d**	**M2d**	**M3d**	**M1d**	**M2d**	**M3d**	**M1d**	**M2d**	**M3d**	**M1d**	**M2d**	**M3d**	**M1d**	**M2d**	**M3d**
Mix	−0.00	−0.04	−0.04	−0.10	*−0.22*	−0.20	0.07	0.06	0.06	−0.10	−0.13	−0.11	−0.06	−0.15	−0.16	*−0.12*	**−0.13**	**−0.13**
	(0.04)	(0.04)	(0.04)	(0.11)	(0.11)	(0.11)	(0.10)	(0.10)	(0.10)	(0.10)	(0.10)	(0.10)	(0.10)	(0.10)	(0.10)	(0.05)	(0.05)	(0.05)
Switch	0.05	0.01	0.03	−0.03	−0.14	−0.11	0.04	0.03	0.03	−0.03	−0.11	−0.14	0.23	0.19	0.16	−0.01	−0.04	−0.04
	(0.04)	(0.04)	(0.04)	(0.11)	(0.11)	(0.11)	(0.08)	(0.08)	(0.08)	(0.10)	(0.11)	(0.11)	(0.14)	(0.14)	(0.14)	(0.05)	(0.05)	(0.05)
2.5	0.02	−0.05	−0.03	−0.02	−0.13	−0.11	−0.04	−0.09	−0.08	−0.08	*−0.20*	*−0.20*	−0.02	0.01	0.00	−0.04	−0.12	−0.10
	(0.04)	(0.04)	(0.04)	(0.08)	(0.08)	(0.08)	(0.10)	(0.10)	(0.10)	(0.09)	(0.10)	(0.10)	(0.12)	(0.12)	(0.11)	(0.06)	(0.07)	(0.07)
Maj.	0.03	**−0.08**	*−0.07*	−0.04	**−0.26**	**−0.25**	0.01	*−0.18*	*−0.17*	0.01	−0.16	−0.18	0.07	0.02	0.02	−0.02	**−0.15**	**−0.14**
	(0.03)	(0.03)	(0.03)	(0.07)	(0.07)	(0.07)	(0.07)	(0.08)	(0.08)	(0.08)	(0.10)	(0.10)	(0.04)	(0.04)	(0.04)	(0.03)	(0.04)	(0.04)
Family controls		x	x		x	x		x	x		x	x		x	x		x	x
School controls			x			x			x			x			x			x
*N*	4,254	4,254	4,254	4,947	4,947	4,947	4,259	4,259	4,259	1,582	1,582	1,582	1,530	1,530	1,530	1,999	1,999	1,999

*Multilevel linear probability models; reference category: speaking mainly L1, second generation (and majority); all models control for gender, grade, and language of the school; M2 additionally controls for country of origin and parental SES; M3 additionally controls for proportion foreign language migrant origin in school, mean SES in school, perception of co-operation and sense of belonging at both individual and school levels (see Table 7); bold p < 0.01, italics p < 0.05*.

**Table 7:** School-related predictors of reading scores (upper panel) and educational expectations (lower panel).

**Dep. var. reading scores**	**Austria**	**Belgium**	**Denmark**	**Germany**	**Luxembourg**	**Switzerland**
	**M3a**	**M3a**	**M3a**	**M3a**	**M3a**	**M3a**
Proportion foreign language immigrants in school	**79.87**	**99.63**	19.62	−34.97	42.90	*40.84*
	(14.74)	(17.47)	(17.41)	(27.06)	(30.11)	(19.85)
School mean SES	**91.17**	**80.30**	**21.28**	**65.66**	**50.77**	**64.32**
	(5.65)	(5.23)	(5.63)	(7.85)	(8.14)	(7.39)
Perception of cooperation at school	**4.57**	2.15	**5.03**	1.75	**4.19**	**5.52**
	(1.07)	(1.21)	(1.43)	(1.90)	(1.47)	(1.59)
Sense of belonging to school	1.48	−0.93	−0.26	−0.29	**5.77**	*3.26*
	(0.85)	(1.05)	(1.16)	(1.76)	(1.42)	(1.48)
School level: perception of co-operation	**40.27**	*21.52*	*12.99*	**31.04**	−15.63	13.68
	(6.63)	(9.11)	(6.33)	(7.94)	(18.32)	(7.64)
School level: sense of belonging	**21.72**	16.73	*14.27*	*24.91*	36.24	*19.96*
	(7.10)	(10.16)	(6.37)	(10.96)	(22.58)	(9.43)
*N*	5,027	6,789	5,745	2,076	4,199	3,290
**Dep. var. educational expectations**	**Austria**	**Belgium**	**Denmark**	**Germany**	**Luxembourg**	**Switzerland**
	**M3c**	**M3c**	**M3c**	**M3c**	**M3c**	**M3c**
Proportion foreign language immigrants in school	**0.51**	**0.74**	0.16	*0.24*	0.23	**0.41**
	(0.06)	(0.08)	(0.08)	(0.11)	(0.15)	(0.09)
School mean SES	**0.38**	**0.34**	**0.07**	**0.22**	*0.09*	**0.32**
	(0.02)	(0.02)	(0.02)	(0.03)	(0.04)	(0.03)
Perception of cooperation at school	−0.00	−0.00	0.01	0.01	0.00	0.01
	(0.01)	(0.01)	(0.01)	(0.01)	(0.01)	(0.01)
Sense of belonging to school	0.01	*0.01*	**0.02**	0.01	**0.03**	0.00
	(0.00)	(0.01)	(0.01)	(0.01)	(0.01)	(0.01)
School level: perception of co-operation	*0.07*	−0.02	0.03	0.04	−0.00	0.03
	(0.03)	(0.04)	(0.03)	(0.03)	(0.09)	(0.04)
School level: sense of belonging	0.03	*0.12*	*0.06*	−0.01	0.19	−0.01
	(0.03)	(0.05)	(0.03)	(0.05)	(0.11)	(0.05)
*N*	5,027	6,789	5,745	2,076	4,199	3,290

*See notes for Tables 5 and 6; bold p < 0.01, italics p < 0.05*.

The results from M1a indicate that in all the analyzed countries apart from Denmark, children of immigrants who have switched to the L2 at home outperform those who mainly speak the L1 at home with their parents in terms of reading scores (Table 4). Children who mix both languages do not differ significantly from the mainly L1 speakers, with the exception of Denmark where their reading scores are lower than those of mainly L1 speakers. The inclusion of family level determinants (M2a) tends to cut these differences between the mainly L1 and L2 speakers by between approximately a third and a half, slightly less in Belgium. Taking into account the school-related factors (M3a) has hardly any effect on these differences.

When we focus on the results among the Turkish and ex-Yugoslav subgroup, the results are much more imprecise due to the small size of some of the groups involved in these comparisons. Nevertheless, based on the size of the estimates (rather than their statistical significance), the results described above for all children of immigrants also apply to this subgroup, although family level determinants do not always explain quite as much. Based on the results from Model 3b, the difference between the mainly L1 and the L2 speakers is largest in Germany and Luxembourg, and it is practically non-existent in Denmark.

Analyzing these two groups separately (Supplementary Tables) does not change these conclusions radically. Nevertheless, it should be noted that in Germany the difference between the two language groups is much larger for those with origins in Former Yugoslavia than in Turkey, with a similar tendency in Austria and Switzerland. On the other hand in Denmark, while the difference is not significant in either group, among those with origins in Former Yugoslavia, those who mainly speak the L1 outperform those who speak the L2.

In terms of math scores (Supplementary Tables), the differences between the language groups are substantially smaller. A statistically significant advantage for the L2 speakers is still evident in Austria and Luxembourg and a disadvantage relative to the mainly L1 speakers for those who speak both L1 and L2 in Denmark. These differences are reduced when family level controls are introduced and the latter two differences become statistically insignificant, leaving the difference in Austria as the only statistically significant one. In the selected subgroup, the difference between the mainly L1 speakers and the L2 speakers is quite sizeable in Germany and Luxembourg (same as for reading), though it is not statistically significant for Luxembourg in any of the models, nor for Germany in the final model.

Moving to educational expectations (Table 6), we see substantially fewer differences between the language groups. In model 1c, young people who have switched to L2 have lower expectations than their peers who continue to speak mainly L1 at home in Switzerland. In Germany and Switzerland, young people who mix both languages hold lower expectations than those who speak mainly the L1. Taking into account family factors (Model 2c) tends to increase these differences and they remain quite stable when taking into account school-related factors (Model 3c). In addition, after controlling for other factors young people who speak mainly the L1 at home have higher educational expectations than their peers who switch to L2 or who mix both languages in Denmark.

Turning to the Turkish and ex-Yugoslav subgroup, there are no statistically significant differences between those who speak mainly the L1 and those who speak the L2 with parents, although in Luxembourg there are indications of higher educational expectations of those switching to L2. Lower educational expectations for those mixing the two languages can also be found in Switzerland, with some indications also in Belgium, Germany and Luxembourg. Separating between the two groups (Supplementary Tables) does not change these main results.

Finally, we turn to the measures related to the school, both in terms of composition and school climate. Table 7 shows these results (results from same Models 3a and 3c as in Tables 5, 6). As expected, peer composition in terms of parental SES is associated with both higher reading (and math) scores and higher educational expectations in all countries. In a number of countries, a larger proportion of foreign-language immigrant-origin students is also associated with higher reading scores and educational expectations (controlling for other relevant factors). However, this association is not significant in Denmark and Luxembourg for either of these outcomes (as well as math), nor in Germany for reading score (where the association is negative though not statistically significant for math scores).

At the individual-level, a perception of co-operation among the student body is associated with higher reading scores in Austria, Denmark, Luxembourg and Switzerland (Model 3a), but not with educational expectations in any country (Model 3c). At the school-level, there is also a substantial positive association with reading scores in all countries except for Luxembourg, and the association is not statistically significant in Switzerland. Again, there doesn't seem to be an association with educational expectations, except in Austria. An increased sense of belonging to school at the individual level seems to be positively associated with higher reading scores only in Luxembourg and Switzerland (Model 3a). It is also associated with higher educational expectations in Belgium, Denmark and Luxembourg (Model 3c). Again, the association at the school-level is more widespread, with a substantial positive association in all countries, albeit not statistically significant in Belgium and Luxembourg (Model 3a). In Belgium and Denmark, it is also positively associated with educational expectations and with a relatively large, though not statistically significant, association in Luxembourg.

We also explored interactions between our main independent variables of interest to examine patterns of moderation. However, these were mostly insignificant and unsystematic. Therefore, the results suggest that the explanatory variables related to the family and school are associated equally strongly with the educational outcomes of the three language groups under study. Nevertheless, when examining children of immigrants without the majority in the sample (in the Supplementary Tables), the results for school-related measures were not always in line with those of the sample as a whole. This sample restriction does not change the results related to the differences between language groups substantially though. Overall, whether the school composition and its climate has a differential impact for children of immigrants in comparison with the majority remains a fruitful area for further research.

## Discussion

In this article we have examined both how family and school-related characteristics are associated with patterns of language use at home among children of immigrants, and how these patterns are, in turn, related to educational outcomes in terms of both learning (particularly reading) and expectations. In addition, we have examined how the family and school-related factors confound (or in some cases mediate) these latter associations. In order to examine country differences more closely, we have analyzed children of immigrants with origins in Turkey and the former Yugoslavia more closely, since they are present in significant numbers in a variety of European countries. However, our results related to country differences are tentative since they are based on relatively small numbers of observations.

Our results suggest that higher parental socioeconomic status is related to a lower likelihood to fully maintain the L1 at home and a greater likelihood to fully switch to the L2 in all countries. The highest propensity to switch to L2 was found in Denmark, followed by Germany. This may be partly explained by the fact that in Denmark, there is no mother tongue instruction in languages that are not EU-languages (Timm and Kristjánsdóttir, 2011). Moreover, Denmark and Germany (as well as Austria) are monolingual countries whereas the other countries in focus are multilingual. Linguistic diversity at the school level was also found to be connected with language maintenance (more strongly with mainly L1 but also with L1 together with L2). This might be supported with the previous finding that school safety is associated with higher levels of students' belief in self, consisting of self-efficacy, persistence, and self-awareness (Storlie and Toomey, 2020), if we assume that a diverse school community creates safe and accepting space for linguistic diversity and maintenance of languages (see also Cummins, 2001; Heikamp et al., 2020). Furthermore, previous research has shown that linguistic diversity also benefits minority language proficiency (Seuring et al., 2020). In addition, a higher school socioeconomic composition was also found to be associated with speaking mainly the L1 with parents, which may potentially reflect a greater acceptance of diversity among the teachers or the students.

Among the group of students with origins in Turkey and Former Yugoslavia, we found L1 maintenance (in particular mainly speaking the L1 but also speaking both the L1 and L2 with parents) to be most clearly related to lower reading and math scores in Germany. Nevertheless, this is not just the case in Germany; across most countries there are indications that both continuing to speak mainly the L1 at home as well as speaking both the L1 and L2 are associated with lower learning outcomes, particularly related to reading, Since there are positive findings advocating for the importance of L1 development in school context in order to support school outcomes more generally (e.g., Edele and Stanat, 2016; Ganuza and Hedman, 2018; Cummins, 2021), this suggests that there is a need for more attention to be paid to the possibilities for all children to develop their L1s in the school context at the same time as they develop their skills in the language of instruction. The importance of supporting multilingualism and children of immigrants speaking another language at home is particularly acute for the first generation and among them, young people arriving at a later age. They are the ones who are the most likely to speak another language at home as in many cases their parents are also only learning the new language themselves (cf. Mouw and Xie, 1999). They may also face a number of other challenges in integrating into their new schools and support for multilingualism may ease these transitions. In particular, this is important for refugee children for whom a safe and supportive schooling environment is essential to ensure optimal learning and integration.

Our findings suggest that mainly using the L1 at home may be positively associated with educational aspirations in Denmark, Germany and Switzerland, although in the Danish case this does not hold for the two origin groups that we focus on. The results lend some support to the idea that L1 use may be a way for families to transmit high parental educational aspirations to their children. However, this may not always translate all the way from educational aspirations to realistic expectations (Friberg, 2019). Moreover, since we do not see the association between language use and educational expectations as being related to the stratification of the educational system, this lends further support to previous research arguing that so-called immigrant optimism is not related to a lack of information about educational systems (Salikutluk, 2016; Tjaden and Hunkler, 2017).

There is growing consensus that the use of L1s in teaching has several advantages. In linguistically responsive teaching, students' multilingualism is viewed as a resource and students are promoted in their ability to draw on their entire linguistic repertoire for learning subject-specific content (Lucas and Villegas, 2013). This means that academic content is learned more efficiently: since prior knowledge may be encoded in languages other than that of instruction, pedagogies that promote the transfer of knowledge from one language to another allow students to build on that prior knowledge and learn faster (Cummins, 2001; García and Hesson, 2015). The goal is thus to achieve better learning outcomes for multilingual students by supporting multilingual practices in classrooms. The term translanguaging has also emerged in particular in the United States to refer to the practice of using one's whole linguistic repertoire for communication and learning purposes (e.g., García and Hesson, 2015). In educational contexts, translanguaging also highlights multilingualism as a persistent feature rather than a transition period in the transition to the majority language.

Importantly, we don't find evidence of the use of both L1 and L2 at home being particularly harmful for reading (or math) scores, with the possible exception of Denmark, despite some teachers' beliefs that children's use of multiple languages may lead to less developed language skills in any of the used languages (Alisaari et al., 2021). However, using both languages seems to be associated with lower educational expectations in at least Belgium and Switzerland, for all children of immigrants analyzed together also Denmark and Germany. One possible explanation for this is that mixing languages with parents may be associated with more family conflict (Tseng, 2020), which may in turn lead to lower educational expectations. It is important to create a safe and supportive space for linguistic diversity and the use of multiple languages side by side in order to support multilingual students' holistic identities (Cummins, 2001), and positively influence their self-esteem and enthusiasm for learning (Catalano et al., 2019).

Yet despite the fact that intercultural education and support for multilingualism, including home languages, is present—and has been present for a long time (e.g., Lange et al., 2010)—in the national curricula and official recommendations of these countries and even at the level of the Council of the European Union (2019) and the Council of Europe (2022), it does not seem to translate into practices that would assist children of immigrants who speak a language other than that of the school at home to reach the same levels of proficiency in reading as their monolingual peers. Thus, there is a clear need for financial resources for education providers and professional development for teachers and school leaders in order to enable the policy recommendations to translate into practices and to provide more equal educational opportunities for all children.

More equal educational opportunities are also supported by cooperative school culture. It is noteworthy that our findings related to school climate are important for all students, not just those with immigrant and minority foreign language backgrounds. When the school culture promotes cooperation and good peer relationships, the learning outcomes tend to be better (see also Yeasmin and Uusiautti, 2018; Heikamp et al., 2020; Kende et al., 2020). School as an institution sends powerful messages on the importance of working together and seeing all group members valuable, and thus, it is important that schools have policies that support collaboration and a positive school climate.

The results are not completely consistent when looking at the students with origins in Turkey and Former Yugoslavia separately from all children of immigrants, For example, Germany is relatively similar to all the other analyzed countries when looking at all students. However, among our focus group, the gap between those speaking mainly the L1 and those speaking only the L2 is substantially larger in Germany than in the other countries. Thus, it is important to look at ethnic groups separately (see also Fan et al., 2011), since children of immigrants are not a homogeneous group. Moreover, paying attention to different country contexts is also relevant since language related gaps are not similar in each context. Previous research has also shown that different educational systems vary in their ability to protect the second-generation youth against the harmful effects of segregation (Baysu and de Valk, 2012). Interestingly, despite the fact that monolingual countries seemed to be associated with higher propensities to switch to speaking the L2 with parents, they are not similarly associated with the gaps between language groups since Denmark represents the other end of the spectrum as Germany here, with Austria in the middle. Denmark is also the least stratified education system among the countries analyzed here. Tentatively it thus seems that greater (and earlier) stratification is not only associated with greater ethnic inequality but also greater learning gaps based on home language use.

A limitation of our study is that it is based on cross-sectional analyses that cannot fully take into account all of the selection processes involved. For example, we cannot assess the reasons for the different patterns of language use: some of this may be related to the reasons for migration (including work migration vs. refugees) and the intention to stay, others to both parents' and children's level of integration (including but not limited to their proficiency in L2) as well as family relationships. Furthermore, we cannot interpret the causality of the results: for example, it might be that the students who maintain their language tend to attend schools in certain areas when the school would not necessarily influence language maintenance. Additionally, with our data, we are not able to assess the students' proficiency in L1, and thus, we cannot make conclusions on the bilingual abilities and their positive effects for these students.

To conclude, a positive school climate is of particular importance in schools with students from vulnerable backgrounds (Berkowitz, 2022). Policies and practices that appreciate cultural and linguistic diversity should be implemented in each school in order to promote cooperation and participation in the school community (see also Borgonovi, 2018). Only in this way can all students experience a strong acceptance of their identities as a whole, including their linguistic resources.

## Data Availability Statement

Publicly available datasets were analyzed in this study. This data can be found at: PISA 2018 database. https://www.oecd.org/pisa/data/2018database/.

## Ethics Statement

Ethical review and approval was not required for the study on human participants in accordance with the local legislation and institutional requirements. Written informed consent from the participants' legal guardian/next of kin was not required to participate in this study in accordance with the national legislation and the institutional requirements.

## Author Contributions

All authors listed have made a substantial, direct, and intellectual contribution to the work and approved it for publication.

## Funding

This research has been funded by the Academy of Finland through an Academy Research Fellowship (Grant No. 316247) and the Finnish Flagships Program (Grant No. 320162).

## Conflict of Interest

The authors declare that the research was conducted in the absence of any commercial or financial relationships that could be construed as a potential conflict of interest.

## Publisher's Note

All claims expressed in this article are solely those of the authors and do not necessarily represent those of their affiliated organizations, or those of the publisher, the editors and the reviewers. Any product that may be evaluated in this article, or claim that may be made by its manufacturer, is not guaranteed or endorsed by the publisher.
